# Genetic predisposition and bioinformatics analysis of ATP-sensitive potassium channels polymorphisms with the risks of elevated apolipoprotein B serum levels and its related arteriosclerosis cardiovascular disease

**DOI:** 10.18632/aging.202628

**Published:** 2021-03-03

**Authors:** Cheng Liu, Tianwang Guan, Yanxian Lai, Junfang Zhan, Yan Shen

**Affiliations:** 1 Department of Cardiology, Guangzhou First People’s Hospital, South China University of Technology, Guangzhou 510180, China; 2 Department of Health Management Center, Guangzhou First People’s Hospital, South China University of Technology, Guangzhou 510180, China

**Keywords:** polymorphism, ATP-sensitive potassium channels, apolipoprotein B, arteriosclerosis cardiovascular disease, exosome-derived microRNAs

## Abstract

Serum concentration of apolipoprotein B (Apo B) is causally associated with arteriosclerosis cardiovascular disease (ASCVD) risk. Whether ATP-sensitive potassium channels (*KATP*) variants predict the risk of increased Apo B concentration (≥ 80 mg/dL) and related ASCVD remain less clear. We recruited 522 subjects with elevated Apo B concentration (≥ 80 mg/dL) and 522 counterpart subjects (< 80 mg/dL) from South China to assess the associations of *KATP* variants (rs11046182, rs78148713, rs145456027 and rs147265929) with the risks of increased Apo B serum concentration (≥ 80 mg/dL), carotid artery stenosis (CAS) ≥ 50% and new-onset ischemic stroke (IS). Our results showed that only *KATP* SNP rs11046182 (GG genotype) was associated with increased risk of Apo B ≥ 80 mg/dL (adjusted OR=2.17, *P*<0.001) and CAS ≥ 50% (adjusted OR=2.63, *P*=0.011). After median 50.6-months follow-up, subjects carrying GG genotype of rs11046182 were associated with higher risk of new-onset IS (adjusted HR=2.24, *P*=0.024). Further, the exosome-derived microRNAs (exo-miRs) expression profile was identified by next-generation sequencing. 41 exo-miRs were significantly differentially expressed under cross-talk status between high Apo B level (≥ 80 mg/dL) and *KATP* rs11046182. Our study demonstrated that *KATP* variant rs11046182 was associated with higher risks of elevated serum Apo B levels and its related ASCVD, and the possible mechanism was related to specific exo-miRs expression profile of *KATP* rs11046182.

## INTRODUCTION

Apolipoprotein B (Apo B) is the major protein constituent of low-density lipoprotein cholesterol (LDL-C). Increased serum level of LDL-C is recognized to be an independent risk factor for atherosclerotic-related events [1], such as carotid arteriosclerosis stenosis (CAS) ≥ 50% and ischemic stroke (IS). According to the amount of cholesterol ester in the core of LDL-C particle, LDL-C particles are different in sizes and densities such as small, intermediate and large dense LDLs, containing one molecule of Apo B per LDL-C particle regardless of its size. Although in most cases, the independent risk factor for atherosclerotic-related events is not smaller LDL particles, subjects with more of this type of LDL particles will be at higher risk for future cardiovascular events. However, epidemiological studies still fail to distinguish the relative atherogenicity caused by different sizes of LDL particles. This suggests that it is not the composition (sizes and densities) of LDL-C particles, but its quantity (Apo B content) that is the key factor of atherosclerosis. Smaller LDL particles are more likely to enter the arterial wall, and are more susceptible to oxidation, which is essentially as a result of the conformation-changing of Apo B with decreasing LDL particles size. For most individuals, the serum level of Apo B is largely concordant with that of LDL-C, and adds minor effect to LDL-C based risk assessment. However, in a subgroup with inconsistence between the serum levels of Apo B and LDL-C, there is a redundant risk is related to an excess risk of atherosclerotic cardiovascular disease (ASCVD) [2]. On the other hand, statins can control LDL-C levels, but a residual risk of ASCVD still remains, related to high Apo B levels, especially in people with obesity, metabolic syndrome or (and) diabetes [3]. In statin-treated patients, Apo B levels rather than LDL-C levels indeed better predict subsequent ASCVD events. Hence, Apo B levels are more closely associated with ASCVD than LDL-C levels, are the principal drivers of this process, and may be demonstrably a better biomarker for assessing potential ASCVD risk. Statin therapy is linked to decreases risk of ASCVD events, but the profits could be declined by inherent genetic risk. Thus, it is a promising public approach for early ASCVD prevention based on earlier genetic assessment of subjects at increased risk of higher Apo B serum concentrations.

The ATP-sensitive potassium channels (*KATP*) plays as essential well-fidelity metabolic sensors, and also as an important end effector of ischemic preservation, indicating that *KATP* couples metabolic abnormalities to protection against ischemic-related injury. This also emphasizes *KATP* as novel targets for prevention and treatment of ASCVD. The structure of *KATP* is large heteromultimeric protein complex, consisted of four inwardly-rectifying potassium channel subunits (pore-forming subunits, Kir6.x) and four sulfonylurea receptor subunits (regulatory subunits, SURx). The Kir6.x pore-forming subunits are encoding respectively by KCNJ8 (Kir6.1) and KCNJ11 (Kir6.2) (chromosomal mapping to 12p12.1) while SURx regulatory subunits are respectively by ABCC8 (SUR1) and ABCC9 (SUR2) (chromosomal mapping to 11p15.1). The subunit constitution of *KATP* possibly remodel with different physiological and pathological circumstances, involving in substitute splicing of these coding genes as mentioned above, which can result in different subunits being functional in different status. The *KATP* has extremely high genetic diversity. *KATP* mutations were not only correlated with serum lipid disorder [*e.g.*, triglyceride (TIRG), total cholesterol (TC), LDL-C or (and) high-density lipoprotein cholesterol (HDL-C)] [4–6] and ASCVD [7, 8] but also exhibited ethnic and geographical heterogeneity (*e.g.*, Europeans, Africans or East Asians). Nevertheless, the associations of *KATP* mutations with Apo B serum level and its related ASCVD in China are still unclear. Theoretically, the relationship shows the characteristics of ethnic-specific genetic pleiotropy [9] but there may be a mutual genetic basis between lipid disorder and ASCVD [10].

The occurrence and development of elevated Apo B serum concentration and its related ASCVD arises from complex interaction between genetic and environment factors. The exosome-derived microRNAs (exo-miRs) are one of the main classes of non-coding RNAs, and play a critical role as bridge that links genetic and environment factors. Exosomes are important extracellular vesicles with lipid bilayer membrane, and carry cell-specific medium for mediating intercellular communication, especially microRNAs (miRs). The miRs are a class of small (about 22-25 nucleotides long) and endogenous single-stranded RNAs, with an established function of regulating genes at transcriptional and post-transcriptional steps. The exo-miRs take part in almost every physiological or pathological processes ranging from elevated Apo B level to ASCVD. However, the circulating expression profile of exo-miRs and its effect in the process from genotype (*KATP* variants) to phenotype (elevated Apo B serum levels) remain elusive. In present study we investigate possible associations of *KATP* variants with the risks of increased Apo B serum levels (≥ 80 mg/dL) and ASCVD (*e.g.*, CAS ≥ 50% and new-onset IS) in South China, and identify the plasma expression profile of exo-miRs among subjects under specific genotype (*KATP* variants)-phenotype (Apo B ≥ 80 mg/dL) correlations.

## RESULTS

### Clinical baseline characteristics of participants

Participants with or without higher Apo B serum concentration (≥ 80 mg/dL) showed significant differences on serum concentration of TRIG, TC and LDL-C (all *P*<0.001), as shown in Table 1. After a median follow-up of 50.6-months, there was no significant difference on NYHA functional classification between the two groups as well as combined medication, including antiplatelet drugs, beta-receptor blockers (BBs), calcium channel blockers (CCBs), digoxin, diuretics, hypoglycemic agents, mineralocorticoid receptor antagonists (MRAs), nitrates, renin-angiotensin system inhibitors (RSIs), statins, and warfarin (all *P*>0.05), as shown in Table 2.

**Table 1:** Baseline characteristics of study subjects.

	**Apo B ≥ 80 mg/dL (N/%)**		***P* value**
	**NO**	**YES**	
N	522	522	-
Male: Female	406:116	395:127	0.420
Age (Y)	64.9±11.5	63.8±10.4	0.121
Smoking (%)	264(50.6)	282(54.0)	0.265
Drinking (%)	68(13.0)	82(15.7)	0.217
SBP (mmHg)	137.9±22.1	138.9±23.5	0.452
DBP (mmHg)	77.6±14.1	78.5±12.1	0.266
BMI (kg/m^2^)	24.6±4.5	24.7±3.7	0.813
**Medical condition**			
EH (%)	332(63.6)	324(62.1)	0.608
CHD (%)	426(81.6)	406(77.8)	0.124
T2D (%)	268(51.3)	257(49.2)	0.496
AF (%)	16(3.1)	22(4.2)	0.321
**Blood biochemical index**			
TRIG (mmol/L)	1.39±0.91	1.69±0.86	<0.001
TC (mmol/L)	3.54±0.85	5.02±1.09	<0.001
HDL-C (mmol/L)	1.10±0.29	1.09±0.27	0.848
LDL-C (mmol/L)	1.82±0.55	2.86±0.78	<0.001
ApoA1 (mg/dL)	103.0±24.2	103.4±18.9	0.758
WBC (×10^9^/L)	8.47±2.79	8.44±2.98	0.852
HGB (g/L)	132.4±18.5	131.6±17.6	0.467
PLT (×10^9^/L)	233.1±52.5	234.2±66.2	0.780
FBG (mmol/L)	5.54±1.40	5.66±1.32	0.182
P2hBS (mmol/L)	8.73±2.71	9.07±2.98	0.053
HbA1C (%)	5.9±1.1	6.0±1.3	0.183
Scr (μmol/L)	93.0±44.3	90.1±33.0	0.911
BUN (mmol/L)	5.76±2.17	5.74±1.952	0.247
UA (μmol/L)	406.0±113.6	415.9±108.6	0.150
ALT (U/L)	27.9±31.5	30.3±27.3	0.180
AST (U/L)	47.6±74.9	49.2±58.8	0.698
Alb (g/L)	37.2±3.9	37.4±4.0	0.632
Na^+^ (mmol/L)	140.5±3.1	140.3±3.2	0.324
K^+^ (mmol/L)	3.74±0.41	3.73±0.39	0.452
HsCRP (mg/L)	11.6±18.7	13.9±22.6	0.076
ACE (U/L)	32.8±19.7	35.1±22.6	0.076
Renin (pg/mL)	24.7±27.8	26.3±29.6	0.390
Ang I (ng/L)	2.23±1.69	2.08±1.55	0.146
Ang II (ng/L)	63.5±89.2	71.9±101.7	0.157
ALD (ng/L)	181.0±121.4	178.8±117.4	0.771
**Echocardiography**			
**RVD (cm)**	1.74±0.18	1.75±0.19	0.230
**RAD (cm)**	3.36±0.35	3.34±0.28	0.494
**LVD (cm)**	4.83±0.53	4.78±0.59	0.145
**LAD (cm)**	3.08±0.59	3.14±0.53	0.114
**LVEF (%)**	56.8±9.7	56.5±9.1	0.675

**Table 2:** Partial baseline characteristics of study participants at the end of the follow-up.

	**Apo B ≥ 80 mg/dL (N/%)**		***P* value**
	**NO**	**YES**	
**Sample(N)**	**522**	**522**	**-**
**NYHA**			
I	140(43.8)	162(50.6)	0.053
II	157(49.1)	134(41.9)	
III	21(6.5)	16(5.0)	
IV	2(0.6)	8(2.5)	
**Combined medication**			
(A) Antiplatelet drugs	298(93.1)	300(93.8)	0.750
(B) Warfarin	10(3.1)	17(3.3)	0.169
(C) Statins	296(92.5)	297(92.8)	0.880
(D) RSIs	205(66.1)	221(69.1)	0.431
(E) BBs	222(69.4)	210(65.6)	0.311
(F) MRAs	67(20.9)	74(23.1)	0.504
(G) CCBs	76(23.8)	91(28.4)	0.177
(H) Diuretics	80(25.0)	88(27.5)	0.472
(J) Digoxin	30(9.4)	30(9.4)	1.000
(K) Nitrates	49(15.3)	41(14.1)	0.655
(L) Hypoglycemic agents	152(47.5)	179(53.1)	0.155

### Association between *KATP* SNPs and increased Apo B serum concentration (≥ 80 mg/dL)

Only *KATP* rs11046182 was correlated with higher risk of elevated serum Apo B concentration (≥ 80 mg/dL) (GG genotype, adjusted OR=2.17, 95% CI: 1.55-3.05, *P*<0.001), as shown in Table 3.

**Table 3:** Association of *KATP* SNPs with elevated Apo B levels (≥ 80 mg/dL) in study subjects.

**KATP SNPs**		**Apo B ≥ 80 mg/dL (N/%)**		**χ2**	***P* value**	**Cude** **OR (95% CI)**	**Cude** ***P* value**	Adjusted OR (95% CI)^*^	Adjusted *P* value^*^	**Adjusted OR (95% CI)**^#^	**Adjusted *P* value**^#^
		**NO**	**YES**								
*rs11046182*	*G*	298(57.1)	345(66.1)	8.944	0.003	1.47(1.14-1.88)	0.003	1.52(1.17-1.98)	0.002	2.17(1.55-3.05)	<0.001
	*AA+GA*	224(42.9)	177(33.9)	1.00		1.00		1.00	
*rs78148713*	*CC+CT*	30(5.7)	14(2.7)	6.074	0.014	0.45(0.24-0.86)	0.016	0.46(0.24-0.90)	0.022	0.90(0.39-2.10)	0.811
	*T*	492(94.3)	508(97.3)	1.00		1.00		1.00	
*rs145456027*	*CC+CT*	14(2.7)	10(1.9)	0.682	0.409	1.00		1.00		1.00	
	*T*	508(97.3)	512(98.1)	1.41(0.62-3.21)	0.411	1.82(0.79-4.23)	0.162	1.37(0.44-4.29)	0.585
*rs147265929*	*GG+GT*	32(6.1)	46(8.8)	2.716	0.099	1.00		1.00		1.00	
	*T*	490(93.9)	476(91.2)	0.68(0.42-1.08)	0.101	0.64(0.40-1.05)	0.078	0.60(0.32-1.13)	0.114

*Model 1: After adjustment for gender, age, smoking, drinking, WBC, BMI, EH, T2D, liver function (ALT, AST and Alb), renal function (Scr, BUN and UA), HsCRP, HbA1C, HCY, and RAAS activity (ACE, renin, Ang I, Ang II and ALD).

^#^Model 2: It is the same as Model 1, and including dyslipidemia (TRIG, TC, LDL-C, HDL-C and Apo AI).

### Association between *KATP* SNPs and CAS ≥ 50%

*KATP* rs11046182 was also correlated with elevated CAS ≥ 50% risk (GG genotype, adjusted OR=2.63, 95% CI: 1.25-5.54, *P*=0.011) while rs78148713 and rs147265929 were not (*P*=0.917 and 0.360, respectively), as shown in Table 4. In addition, the OR value of increased CAS ≥ 50% risk for *KATP* rs145456027 will not be estimated due to possible bias.

**Table 4:** Association of *KATP* SNPs with CAS ≥ 50% in study participants.

**KATP SNPs**		**CAS ≥ 50% (N/%)**		**χ2**	***P* value**	**Cude** **OR (95% CI)**	**Cude** ***P*-value**	**Adjusted** OR (95% CI)^*^	Adjusted *P* value^*^	**Adjusted OR (95% CI)**^#^	**Adjusted *P* value**^#^
		**NO**	**YES**								
*rs11046182*	*G*	605(60.9)	38(76.0)	4.610	0.032	2.04(1.05-3.95)	0.035	2.78(1.34-5.77)	0.006	2.63(1.25-5.54)	0.011
	*AA+GA*	389(39.1)	12(24.0)	1.00		1.00		1.00	
*rs78148713*	*CC+CT*	42(4.2)	2(4.0)	0.006	0.938	0.94(0.22-4.02)	0.938	0.88(0.19-4.13)	0.868	0.92(0.19-4.37)	0.917
	*T*	952(95.8)	48(96.0)	1.00		1.00		1.00	
*rs145456027*	*CC+CT*	24(2.4)	0(0.0)	1.236	0.266	-		-		-	
	*T*	970(97.6)	50(100.0)	-	-	-	-	-	-
*rs147265929*	*GG+GT*	74(7.4)	4(8.0)	0.021	0.884	1.00		1.00		1.00	
	*T*	920(92.6)	46(92.0)	0.93(0.32-2.64)	0.884	0.58(0.19-1.79)	0.341	0.59(0.19-1.84)	0.360

*Model 1: After adjustment for gender, age, smoking, drinking, WBC, BMI, EH, T2D, liver function (ALT, AST and Alb), renal function (Scr, BUN and UA), HsCRP, HbA1C, HCY, and RAAS activity (ACE, renin, Ang I, Ang II and ALD).

^#^Model 3: It is the same as Model 1, and including dyslipidemia (TRIG, TC, LDL-C, Apo B, HDL-C and Apo AI).

### Association between *KATP* rs11046182 and new-onset IS

**Figure 1:**
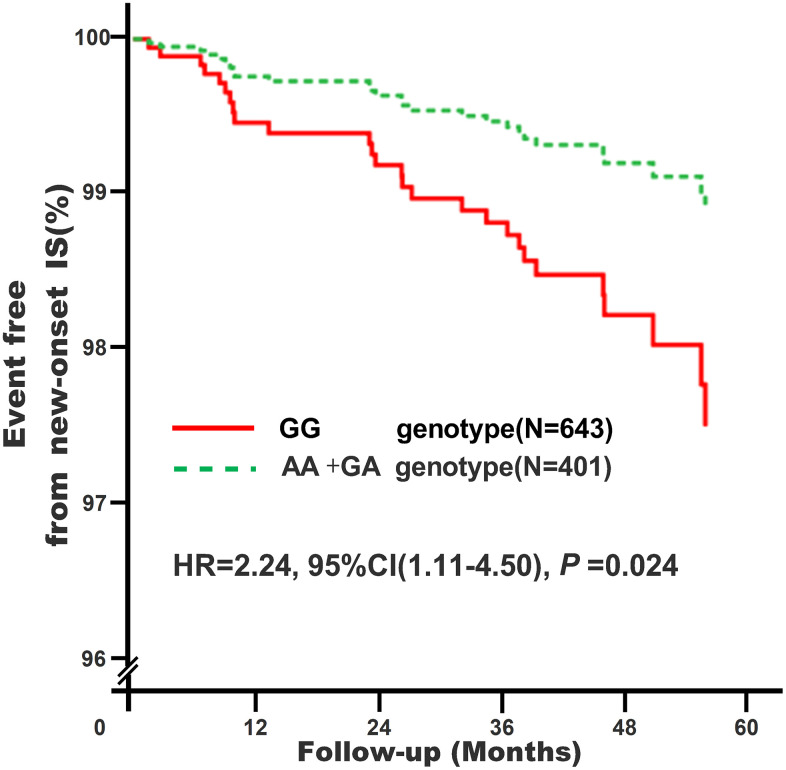
Association of *KATP* rs11046182 with new-onset IS in study subjects^*^. *Model 4: After adjustment for gender, age, smoking, drinking, WBC, BMI, liver function (ALT, AST and Alb), renal function (Scr, BUN and UA), HsCRP, HbA1C, HCY, and RAAS activity (ACE, renin, Ang I, Ang II and ALD), dyslipidemia (TRIG, TC, LDL-C, Apo B, HDL-C and Apo AI), medical condition (EH, CAD, T2D and AF), NYHA functional classification, combined medication (antiplatelet drugs, warfarin, statins, RSIs, BBs, MRAs, CCBs, diuretics, digoxin, nitrates, and hypoglycemic agents) and echocardiography index (RVD, RAD, LVD, LAD, and LVEF).

Subjects carrying GG genotype of *KATP* rs11046182 were correlated with elevated new-onset IS risk (adjusted HR=2.24, 95% CI: 1.11-4.50, *P*=0.024) via a median follow-up of 50.6 months, as shown in Figure 1.

### Clinical characteristics of participants with increased Apo B serum concentration (≥ 80 mg/dL) in plasma exo-miRs expression profiling analyses

As shown in Table 5, no significant differences showed in clinical characteristics (all *P*>0.05) between the two genotypes (AA+GA vs. GG) of *KATP* rs11046182 among subjects with increased Apo B serum concentration (≥ 80 mg/dL).

**Table 5:** Clinical characteristics between different genotypes of *KATP* rs11046182 in subjects with elevated Apo B serum levels (≥ 80 mg/dL) in plasma exo-miRs expression profiling and bioinformatics analysis.

	**Genotypes of KATP rs11046182**		***P* value**
	***AA+GA***	***GG***	
N	5	5	-
Male: Female	3:2	3:2	1.000
Age (Y)	47.8±7.5	48.4±8.8	0.910
SBP (mmHg)	123.4±4.8	125.6±7.9	0.610
DBP (mmHg)	74.4±6.9	76.8±8.8	0.643
BMI (kg/m^2^)	23.5±3.7	23.9±3.2	0.859
TRIG (mmol/L)	1.21±0.22	1.22±0.19	0.973
TC (mmol/L)	3.91±1.24	4.01±0.84	0.876
LDL-C (mmol/L)	2.38±0.46	2.46±0.58	0.803
HDL-C(mmol/L)	1.46±0.25	1.45±0.39	0.955
Apo B (mg/dL)	105.0±22.0	119.6±19.1	0.294
Apo A1 (mg/dL)	152.6±32.4	152.0±28.5	0.976
WBC (×10^9^/L)	7.42±2.76	6.59±4.27	0.727
HGB (g/L)	130.0±19.0	133.6±10.9	0.723
PLT (×10^9^/L)	199.4±49.9	241.4±45.3	0.201
FBG (mmol/L)	4.84±0.52	4.93±0.72	0.827
P2hBS (mmol/L)	6.63±0.90	6.59±0.97	0.948
HbA1C (%)	5.3±0.6	5.5±0.3	0.406
Scr (μmol/L)	84.0±26.0	77.2±26.0	0.690
BUN (mmol/L)	4.88±0.88	5.20±0.69	0.540
UA (μmol/L)	406.0±40.6	385.2±81.4	0.623
ALT (U/L)	21.5±14.1	19.4±5.8	0.763
AST (U/L)	21.4±9.1	21.2±2.4	0.963
Alb (g/L)	37.0±2.6	40.6±3.8	0.123
Na^+^ (mmol/L)	142.4±2.7	141.7±4.2	0.788
K^+^ (mmol/L)	4.17±0.41	4.23±0.42	0.836
HsCRP (mg/L)	9.8±4.0	11.5±6.7	0.642
MAU (ACR*, mg/g)	123.2±49.1	148.6±51.7	0.449
HCY (μmol/L)	11.4±2.2	10.4±2.9	0.531
ACE (U/L)	33.4±24.3	36.9±14.7	0.791
Renin (pg/mL)	22.9±25.7	29.7±36.6	0.742
Ang I (ng/L)	2.06±1.41	2.64±2.28	0.645
Ang II (ng/L)	151.9±114.1	166.1±203.4	0.896
ALD (ng/L)	222.9±60.2	331.4±107.4	0.084

*ACR: urinary albumin-to-creatinine ratio.

### DE exo-miRs under cross-talk status between *KATP* rs11046182 and elevated Apo B serum concentration (≥ 80 mg/dL)

**Figure 2:**
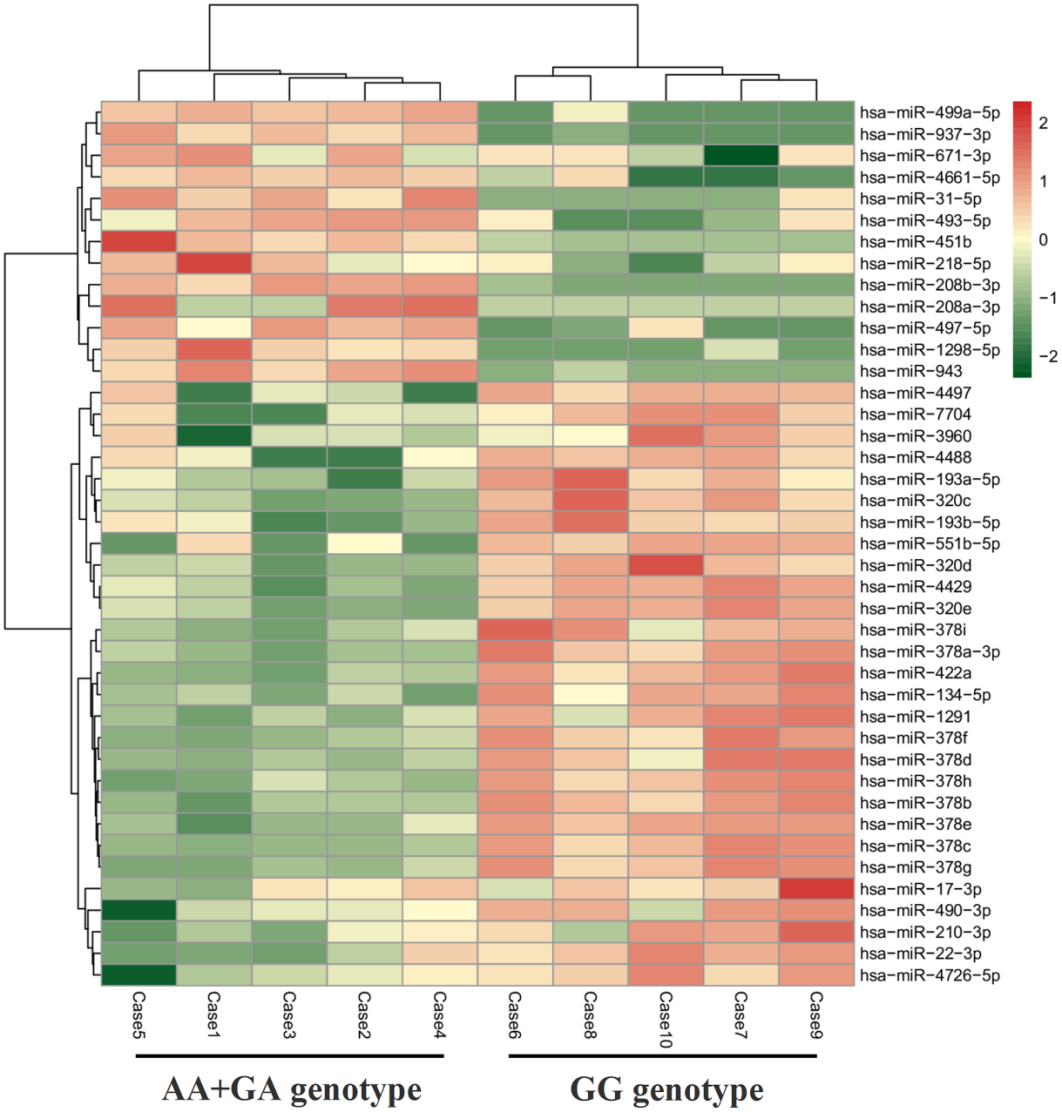
**Heatmap of DE exo-miRs between different genotypes of *KATP* rs11046182 in subjects with elevated Apo B serum levels (≥ 80 mg/dL).**

A total of 615 exo-miRs were detected by implementing strict data quality control. Using reads per million (RPM) values < 10, and *P* < 0.05 as threshold cutoff to exclude the low expression of exo-miRs, 41 exo-miRs were then found to be obviously DE between the two genotypes (AA+GA vs. GG) of rs11046182, as shown in Supplementary Figure 1, Figure 2 and Table 6. Twenty eight exo-miRs were up-regulated in participants with GG genotype of rs11046182 compared to those with A-allele (AA+GA) while 13 exo-miRs were down-regulated, as shown in Table 6. In particular, miR-22-3p had the highest expression level among the 41 DE exo-miRs. The highest up-regulated and down-regulated miRs were miR-320d (5.34-fold changes) and miR-493-5p (5.04-fold changes), respectively. The miR-208a-3p, miR-208b-3p and miR-499a-5p belong to the miR-208 family based on highly homologous sequence. Besides miR-208 family, there were also anther three exo-miRs families as follows: miR-193 (*e.g.*, 193a-5p and 193b-5p), miR-320 (*e.g.*, 320c~320e), and miR-378 (*e.g.*, 378a-3p, 378b~378h). In addition, only 6 of the 41 DE exo-miRs were found to be obviously DE between the two genotypes (AA+GA vs. GG) of rs11046182 in participants with decreased serum Apo B levels (< 80 mg/dL). 2 exo-miRs (miR-31-5p and miR-497-5p) were up-regulated and 4 exo-miRs (miR-320c/d, miR-4429 and miR-134-5p) were down-regulated in subjects carrying GG genotype of rs11046182 compared to those with AA+GA genotype, as shown in Supplementary Figure 2 and Supplementary Table 9. It exhibited opposite expression patterns in subjects with or without increased Apo B serum levels (≥ 80 mg/dL) under the genetic background of *KATP* rs11046182.

**Table 6:** DE exo-miRs between different genotypes of *KATP* rs11046182 in subjects with elevated Apo B serum levels (≥ 80 mg/dL).

	**miR ID**	**Genotypes**		**Fold**	**P value**	**Up/down**
		**AA+GA**	**GG**			
1	hsa-miR-31-5p	1.34	0.24	-2.45	0.004751	down
2	hsa-miR-451b	8.10	0.44	-4.21	0.005722	down
3	hsa-miR-499a-5p	26.82	4.04	-2.73	0.007623	down
4	hsa-miR-671-3p	101.36	19.11	-2.41	0.018733	down
5	hsa-miR-208b-3p	2.42	1.02	-1.25	0.018905	down
6	hsa-miR-937-3p	3.25	1.16	-1.48	0.024341	down
7	hsa-miR-493-5p	82.78	2.522	-5.04	0.025111	down
8	hsa-miR-208a-3p	0.75	0.20	-1.91	0.025430	down
9	hsa-miR-218-5p	76.76	4.22	-4.19	0.029824	down
10	hsa-miR-1298-5p	5.43	1.01	-2.43	0.035482	down
11	hsa-miR-497-5p	1.19	0.32	-1.89	0.041277	down
12	hsa-miR-4661-5p	14.27	5.58	-1.35	0.043491	down
13	hsa-miR-943	1.51	0.24	-2.65	0.049052	down
14	hsa-miR-490-3p	1.46	24.78	4.09	2.50E-05	up
15	hsa-miR-378c	70.22	1822.48	4.70	3.03E-05	up
16	hsa-miR-378g	6.52	59.63	3.19	5.65E-05	up
17	hsa-miR-378f	8.04	107.65	3.74	6.95E-05	up
18	hsa-miR-1291	2.77	20.19	2.87	7.18E-05	up
19	hsa-miR-378e	1.07	23.87	4.48	0.000129	up
20	hsa-miR-378h	0.60	10.85	4.17	0.000132	up
21	hsa-miR-378i	138.95	1124.06	3.02	0.000135	up
22	hsa-miR-378a-3p	2047.51	19589.27	3.26	0.000162	up
23	hsa-miR-320d	245.90	9941.44	5.34	0.000258	up
24	hsa-miR-422a	4.49	46.97	3.39	0.000264	up
25	hsa-miR-378d	45.59	968.59	4.41	0.000309	up
26	hsa-miR-378b	0.70	18.01	4.69	0.000455	up
27	hsa-miR-22-3p	7649.18	25786.91	1.75	0.000498	up
28	hsa-miR-4429	12.16	248.78	4.35	0.001036	up
29	hsa-miR-320e	50.86	955.40	4.23	0.001090	up
30	hsa-miR-4726-5p	0.57	3.75	2.71	0.001247	up
31	hsa-miR-7704	0.77	9.68	3.66	0.001776	up
32	hsa-miR-210-3p	41.14	152.05	1.89	0.002202	up
33	hsa-miR-320c	649.53	12815.27	4.30	0.002883	up
34	hsa-miR-134-5p	497.18	13530.88	4.77	0.003356	up
35	hsa-miR-4488	0.96	6.53	2.77	0.003878	up
36	hsa-miR-3960	1.66	23.22	3.81	0.004081	up
37	hsa-miR-193a-5p	321.15	3274.08	3.35	0.007049	up
38	hsa-miR-551b-5p	0.51	4.36	3.08	0.008227	up
39	hsa-miR-193b-5p	22.09	256.94	3.54	0.023852	up
40	hsa-miR-17-3p	4.74	17.37	1.87	0.024641	up
41	hsa-miR-4497	0.81	4.21	2.38	0.025088	up

### GO analysis of enriched categories

**Figure 3:**
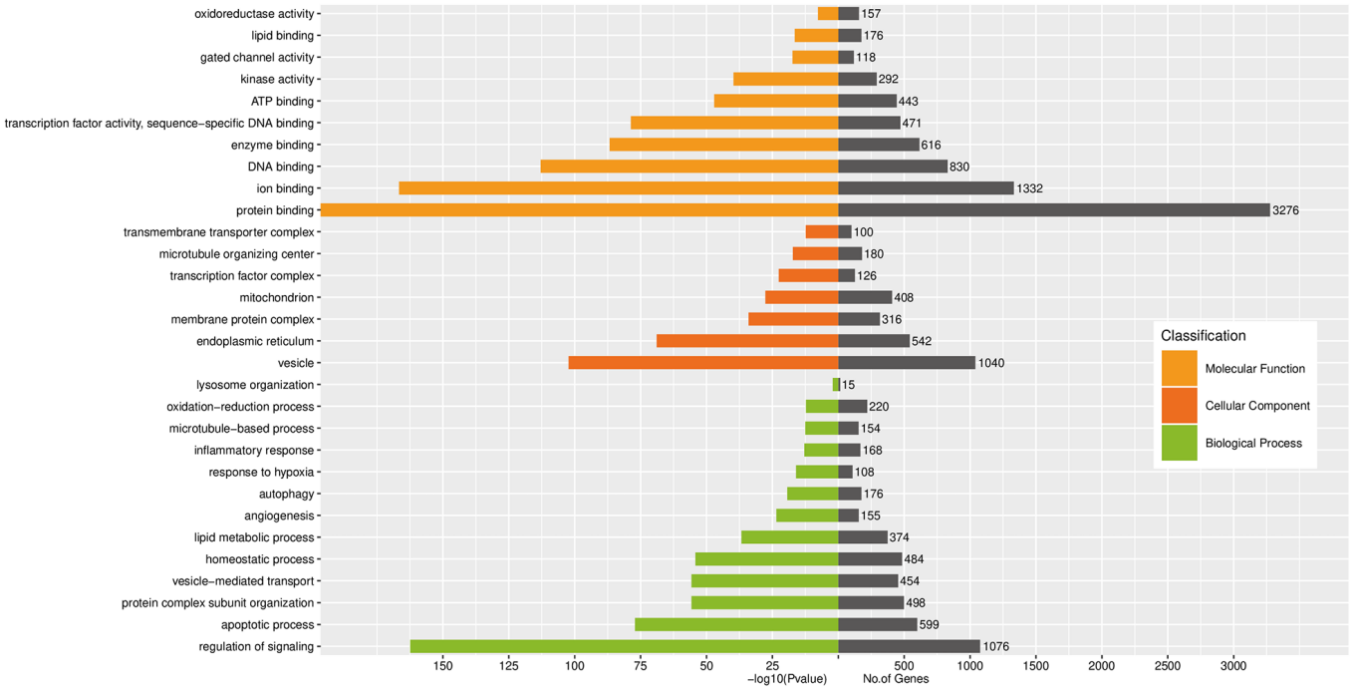
**GO analysis of enriched biological processes, cellular component and molecular functions regulated by CTGs of top 10 DE exo-miRs.**

Three terms of GO enriched categories analysis was carried out for those candidate target genes (CTGs) regulated by the top 10 DE exo-miRs, as shown in Figure 3. The CTGs of DE exo-miRs in high Apo B levels subjects with GG genotype of rs11046182 were obviously associated with the following biological processes: regulation of signaling, apoptotic process, protein complex subunit organization, vesicle-mediated transport, oxidation-reduction/homeostatic/lipid metabolic process, angiogenesis and autophagy so on, involving in the following cellular component such as vesicle, endoplasmic reticulum, mitochondrion and membrane protein/transcription factor/transmembrane transporter complex. Molecular functions affected by predicted target genes of DE exo-miRs, and can be mainly classified into two types: binding regulation (*e.g.*, protein, ion, DNA, enzyme and ATP) and activity regulation (*e.g.*, transcription factor, kinase activity, gated channel and oxidoreductase).

### KEGG analysis of enrichment pathways

**Figure 4:**
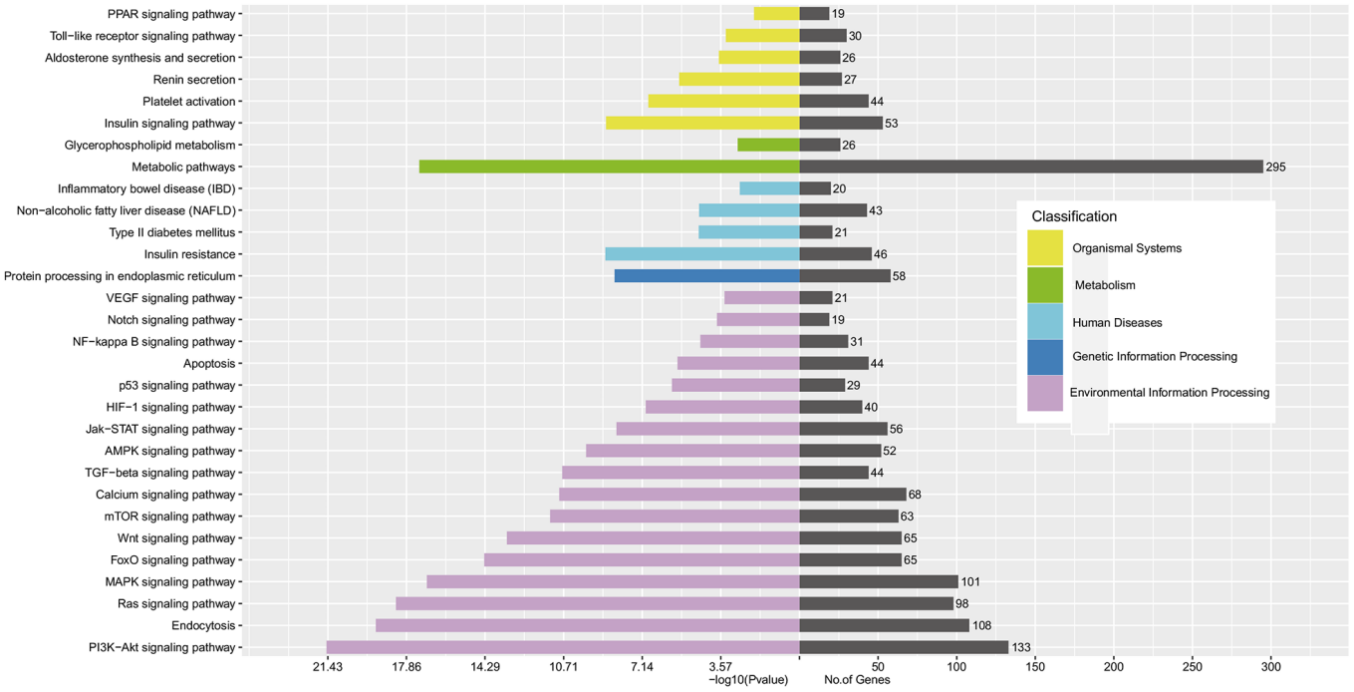
**KEGG analysis of enrichment pathway regulated by CTGs of top 10 DE exo-miRs.**

KEGG analyses of top 10 DE exo-miRs showed that the top 30 enrichment pathways were mainly associated with metabolic pathways, environmental information/genetic information processing (*e.g.*, endocytosis, signaling pathways of PI3K-Akt, MAPK and Ras, and protein processing in endoplasmic reticulum, etc), metabolism-related diseases (*e.g.*, T2D, non-alcoholic fatty liver disease, and insulin resistance, etc), and organismal systems (*e.g.*, insulin signaling pathway, platelet activation, and Toll-like receptor signaling pathway, etc), as shown in Figure 4.

### Target interactome network of top 10 DE exo-miRs

**Figure 5:**
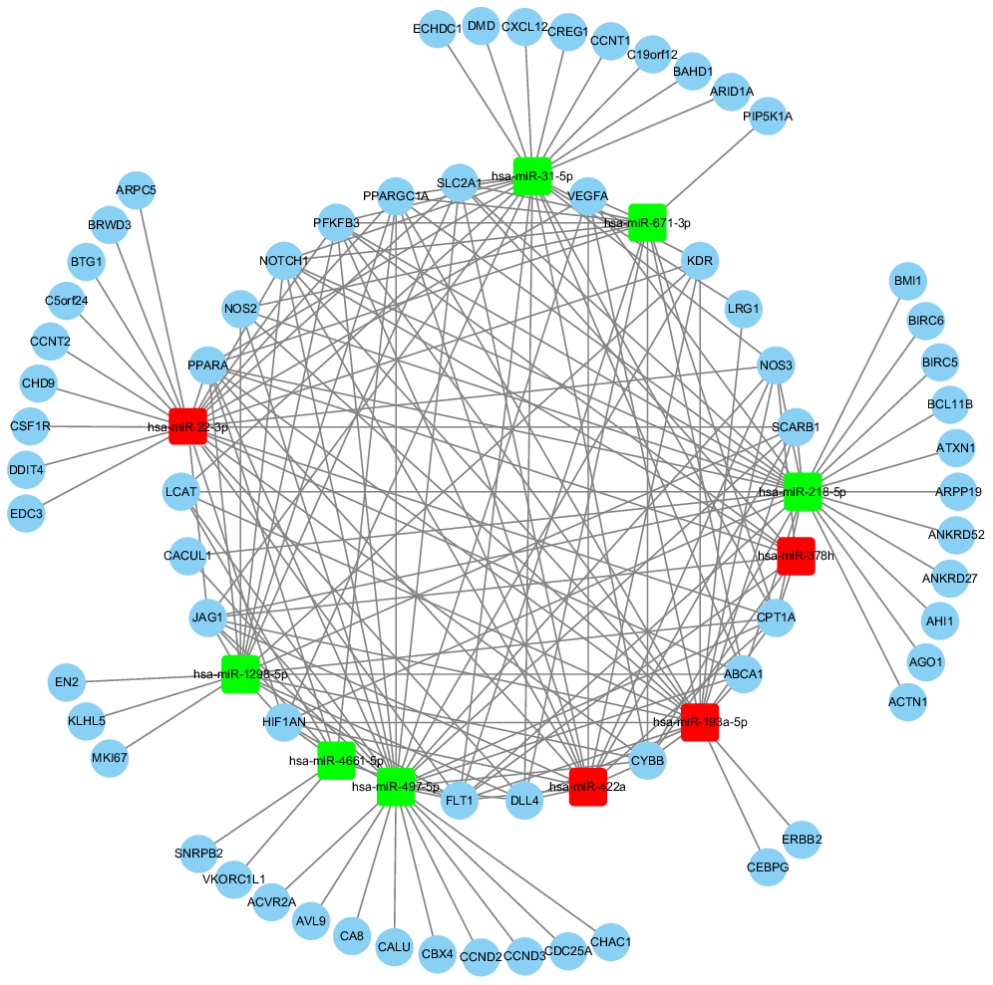
**The cross-talk diagram on miRs-gene and gene-gene from top 10 DE exo-miRs.** **Using combined score > 0.9 as threshold cutoff, 10 exo-miRs and 65 CTGs were included in the internet. Red color represents the up-regulated exo-miRs; Green color represents the down-regulated exo-miRs.

Using combined score greater than > 0.9 as threshold cutoff, a miRs/gene and gene/gene interaction network was consisted of 10 exo-miRs and 65 CTGs, regulated by differently regulated exo-miRs among increased Apo B serum levels (≥ 80 mg/dL) subjects with GG genotype of rs11046182, as shown in Figure 5. The highly correlated CTGs were as follows: ATP binding cassette subfamily A member 1 (ABCA1), carnitine palmitoyltransferase 1A (CPT1A), cytochrome b(558) subunit beta [CYBB, also NADPH oxidase 2 (NOX2)], hypoxia-inducible factor-1alpha (HIF-1α), jagged 1 (JAG1), Notch homolog 1 (translocation-associated) (Notch1), peroxisome proliferator-activated receptor-gamma coactivator-1alpha (PGC-1α), peroxisome proliferator-activated receptor-alpha (PPARα), 6-phosphofructo-2-kinase/fructose-2,6-biphosphatase 2 (PFKFB2), and solute carrier family 2 member 1 [SLC2A1, also called glucose transporter 1 (GLUT1)], etc.

## DISCUSSION

To the best of our knowledge, this is the first comprehensive study to examine the possible associations of *KATP* SNPs with elevated Apo B serum concentration (≥ 80 mg/dL [11]) and ASCVD in south China. The data indicate that the GG genotype of rs11046182 was only linked to increased risk (about increased by 1.17-fold) of elevated Apo B serum levels rather than the other types of serum lipid disorder (*e.g.*, TRIG, TC, LDL-C, HDL-C or (and) Apo AI, Supplementary Tables 3–7). The effects were not related to the other 3 *KATP* variants (*e.g.*, rs78148713, rs145456027 and rs147265929). Interestingly, in this study there was no significant difference on body mass index (BMI), SBP, P2hBS, hypersensitive C-reactive protein (HsCRP) in subjects with or without higher Apo B serum levels (≥ 80 mg/dL) (Table 1), but its average levels of BMI (> 24.0 kg/m^2^), SBP (> 130 mmHg), P2hBS (> 7.8 mmol/L), HsCRP (> 3 mg/L) were higher than normal especially in higher Apo B level group, which was also complicated with higher serum levels of TRIG, TC and LDL-C. On the other hand, subjects with GG genotype of *KATP* rs11046182 were indeed related to higher serum levels of P2hBS and HsCRP besides high Apo B serum levels (Supplementary Table 8). It is suggesting that high Apo B serum level participants with GG genotype of *KATP* rs11046182 sustain a status of metabolic disorders and inflammation. Insulin resistance (IR) acts a major role in the pathogenesis of this process. Indeed, a previous study reported that *KATP* rs5219 was correlated with IR [12]. The loci also act an important role in process of glucose-induced insulin secretion among Turks [13] as well as the other two *KATP* variants (rs1799854 and rs1799859), the effect was not existed in Caribbeans (rs5219) [14] and Poles (rs1799854) [8]. On the other hand, blood lipid disorder is heterogeneous disease characterized by irregular levels of serum lipids and lipoproteins. Our findings are partially accordant with some similar studies. KATP rs1799854 (TT genotype) was linked to higher serum HDL level among non-diabetic patients in Nigeria as well as lower serum levels of TG, TC and LDL [4]. KATP rs1799854 and rs1799859 were linked to increased TRIG serum level among Croatia diabetic patients receiving sulfonylurea treatment [5]. KATP rs5219 (KK+EK genotype) was correlated with higher TC/HDL-C ratio among young Chinese Han people with prediabetes [6]. These findings indicated that KATP rs11046182 could be a latent genetic predisposition marker for elevated Apo B serum levels for Southern Chinese.

Cholesterol-rich, Apo B-containing lipoproteins are now widely accepted as the most important causal agents of ASCVD [15]. CAS no less than 50% is known as a new subtype of ASCVD besides acute myocardial infarction (AMI) and stroke [16]. In this study we further found that subjects carrying with GG genotype (the high Apo B risk genotype) of rs11046182 were also linked to moderate risk (about a 0.63-fold increase) of CAS ≥ 50% at studying enrollment, and further correlated with high risk (about a 1.24-fold increase) of new-onset IS after median follow-up of 50.6-months. These findings are partially accordant with the other related studies that found that *KATP* rs61688134 was associated with AMI among Italians [7] while *KATP* rs1799854 was with stroke in diabetic Polish [8]. These results suggest that *KATP* rs11046182 may be an optimal marker of elevated risk of Apo B related ASCVD.

The interplay between genetic and environment factors causes the development of dyslipidemia and related ASCVD. The circulating exo-miRs, as bridge that links genetic and environment factors, play an essential effect in physiological or pathological processes from elevated Apo B level to ASCVD. However, the expression profile of exo-miRs in biological process from genotype (*KATP* rs11046182) to phenotype (Apo B ≥ 80 mg/dL) is still largely unclear. Our results firstly characterized the circulating expression profile of exo-miRs (Table 4) among increased Apo B level subjects with the two genotypes of *KATP* rs11046182. Synchronously, studies had shown that those DE exo-miRs played a crucial effect in development of arteriosclerosis (*e.g.*, miR-17-3p [17], miR-22-3p [18], miR-490-3p [19], miR-193 family [20], miR-320 family [21] and miR-378 family [22], etc), involving in endothelial cells dysfunction, vascular smooth muscle cells proliferation and migration, plaque angiogenesis, apoptosis, autophagy and macrophage lipid deposition. Many studies showed that miR-31-5p [23], miR-210-3p [24] and miR-208 family [25] were significantly associated with the stenosis degree of atherosclerotic plaques as well as unstable phenotype, suggesting that these exo-miRs could be correlated with the potential cardiovascular events risk. Our findings were in favor of recent observations that reported the DE miRs (*e.g.*, miR-17-5p [26], miR-210 [27], miR-218 [28], miR-422a [29], miR-497 [30], miR-4429 [31], miR-208 family [25], miR-320 family [31] and miR-378 family [32]) in patients with IS, and part of them could be as markers for an early diagnosis of stroke. In a 4-year prospective study for identifying the markers of CAS related IS, Gacon J et al. [25] reported that elevated miR-208b-3p level were obviously linked to cerebral ischaemic events risk. This finding was accordant with the investigation by Jin F et al. [28] who reported that miR-378 and miR-218 were independent predicting factors for the severity in patients with acute IS. Further, the plasma level of miR-210 [33] was related to a worsening prognosis of stroke while miR-17 to future stroke recurrence [34]. Under the genetic condition of *KATP* rs11046182, these exo-miRs (*e.g.*, miR-17-3p, miR-22-3p, miR-31-5p, miR-134-5p, miR-210-3p, miR-490-3p, miR-208 family, miR-320 family and miR-378 family) run through the whole pathological processes from the accumulation of cardiovascular risk factors (*e.g.*, smoking, physical inactivity, unhealthy diet, obesity and aging) to the occurrence of atherosclerotic-related events and even death. In addition, there were another four exo-miRs as follows: miR-1291, miR-4488, miR-4726-5p, and miR-7704. However, the relationships of the 4 exo-miRs with cardiovascular disease are still unknown, needing further research.

To further evaluate the roles of exo-miRs under interaction between genetic and environment factors, GO and KEGG analysis for the top 10 DE exo-miRs related 1156 CTGs were carried out among elevated Apo B ≥ 80 mg/dL subjects with GG genotype of rs11046182. GO analyses (Figure 3) showed that enrichment of CTAs acted pivotal roles in BP, CC and MF, accordant with a regulatory role for these exo-miRs in the processes of transcription and translation [35] on dyslipidemia and related ASCVD. KEGG pathways analyses (Figure 4) showed that metabolic pathways, PI3K-Akt signaling pathway and endocytosis were the three most significant differentially regulated pathways. In particular, PI3K-Akt signaling pathway, which acts a principal role in regulating growth factor signals (*e.g.*, glucose/lipid/protein metabolism, etc) under disease status (*e.g.*, obesity and T2D) [36]. These results suggested that those DE exo-miRs might act a key effect in elevated Apo B serum levels and its induced ASCVD by regulating these 3 pathways, especially PI3K-Akt signaling pathway.

### Strengths and limitations

The major advantage of the research was firstly evaluate the associations of *KATP* mutations with the risk of increased Apo B concentration (≥ 80 mg/dL) and ASCVD in South China, and characterize the circulating expression profile of exo-miRs under interplay status between genetic (KATP variants) and environmental (elevated Apo B serum levels) factors, intimating that the possible epigenetic modification effect of exo-miRs in development of dyslipidemia and its related atherosclerotic vascular events. The major disadvantages of the study were as follows: Firstly, due to the sample size (N=1044), large-scale subgroup analysis based on Apo B serum level (< 80 mg/dL vs. ≥ 80 mg/dL) and genotypes (GG vs. AA + GA) of *KATP* rs11046182 will help to further verify the hypothesis that the occurrence and development of increased Apo B serum concentration and its related ASCVD arises from complex interaction between genetic and environment factors. Secondly, Bonferroni correction was executed to correct significance thresholds, but false-positive results may still occur. Thirdly, a rudimentary bioinformatics analysis was only executed, and the non-specific effect and miss-distance effect may exist, owing to lack of verification at the cellular and molecular levels. Therefore, the results of the study should be interpreted carefully.

## CONCLUSIONS

*KATP* rs11046182 was correlated with increased risks of elevated serum Apo B concentration (≥ 80 mg/dL) and ASCVD, suggesting that this variant is a prospective clinical translational target for precision prevention and early-detection strategies for those disorders, and needs further verification by prospective studies with large sample sizes in different ethnic populations. The potential molecular regulatory may be involved in these significantly DE exo-miRs (especially the top 10 DE exo-miRs) and metabolic related pathways (especially PI3K-Akt pathway) under those cross-talk status, warrant further research.

## MATERIALS AND METHODS

### Study subjects

The ethics approval (K-2017-043-02) of this study was granted from Guangzhou First People’s Hospital, South China University of Technology. The present study was conducted in consistent with the Helsinki Declaration and the ethics guidelines of the institutional. A total of 522 participants with increased Apo B serum levels (≥ 80 mg/dL) and 522 counterpart subjects (< 80 mg/dL) were enrolled to the research from South China. All participants with blood lipid disorder were newly identified referring to guidelines [11] as follows: increased serum concentrations of Apo B (≥ 80 mg/dL), TRIG (≥ 1.7 mmol/L), TC (≥ 5.2 mmol/L) or (and) LDL-C (≥ 1.4 mmol/L), and (or) decreased serum concentrations of apolipoprotein AI (Apo AI) (< 120 mg/dL) and HDL-C (< 1.0 mmol/L). The combined medical conditions including essential hypertension (EH) [37], coronary atherosclerotic heart disease (CAD) [38], atrial fibrillation (AF) [39] or (and) type 2 diabetes mellitus (T2D) [40] were also evaluated referring to relevant guidelines. Potential participants were excluded from the study if they had if they had (1) past history of stroke or transient ischemic attack, (2) Elevated levels (>3 times upper limit of normal) of aspartate aminotransferase (AST) and alanine aminotransferase (ALT), (3) decreased level (<90ml/min•1.73 m^2^) of estimated glomerular filtration rate (eGFR), (4) or (and) any other medical disorders or drugs that could result in kinds of dyslipidemia as mentioned above. Participant’s medical records were assembled via interviewing patient himself and physicians as well as reviewing of medical records. Standard analytical methods were performed to assess blood biochemical indexes on admission to the study. Bilateral carotid ultrasound was executed on enrollment to the study referring to relevant recommendations [41].

### Genotyping assay

Four *KATP* single nucleotide polymorphisms (SNPs) (*e.g.*, rs11046182, rs78148713, rs145456027 and rs147265929) were genotyped with the MassARRAY (Sequenom) system as previously described methods [42]. Primer software (Version 5.0, Cambridge, USA) was used to design the specific primers for the 4 *KATP* variants based on the *KATP* gene sequence information in GenBank (NC_000012.12:g.21768149G>A; NC_000012.12:g.21777582T>C; NC_000012.11:g.21943896T>C; NC_000011.10:g.17391521T>G) (Supplementary Table 1). The specific primers of *KATP* SNPs were composited by Invitrogen (Guangzhou, China). The SNPs determination accuracy was 100% for each variant of *KATP*.

### Endpoint

Primary follow-up end-point was new-onset IS. All stroke subjects were survivors of IS, and determined by magnetic resonance image and/or computed tomography scanning of the brain referring to relevant guidelines [43]. Subjects were recruited to the study on the date of initial evaluation for increased Apo B serum levels (≥ 80 mg/dL) since first medical examination. IS-free event survival time was defined as the time from the enrollment date to the date of initial evaluation for IS or last follow-up. The date of final follow-up was Dec 31, 2019. The median follow-up time was 50.6 (range: 43.5-58.7) months.

### Identification of exo-miRs expression profile

Another total of 10 participants from South China with only elevated serum Apo B concentration (≥ 80 mg/dL) were newly enrolled to the study (the baseline characteristics was shown in Table 5). Participants with other types of serum lipid disorder were ruled out from the study as described above. All participants combined with smoking, drinking, EH, CAD, AF, T2D, IS, abnormal liver/kidney function, or (and) any other medical disorders or drugs that could result in kinds of dyslipidemia were also excluded from the study as described above. Then, the exo-miRs expression profile was analyzed according to our previous method with minor modifications [44], including blood sample collection, isolation exosomes from plasma, extraction RNA from exosomes, exo-miRs sequencing, and analysis of sequencing data (detail information was presented in methods section of Supplementary Material). In particular, exo-miRs sequencing was executed at Ribobio Co. (Guangzhou, China) with Illumina HiSeq2500 with single-end 50bp (Illumina, Carlsbad, USA). 3' adapter sequence is: 5’-AGATCGGAAGAGCACACGTCT-3’. 5' adapter sequence is: 5’-GTTCAGAGTTCTACAGTCCGACGATC-3’. The novel exo-miRs discovery was performed using miRDeep2 based on miRBase21 (http://www.mirbase.org).

### Statistical analysis

The SPSS software (version 24, SPSS Co., USA) was used for statistical analyses. The Hardy-Weinberg equilibrium was evaluated for control subjects as shown in Supplementary Table 2. Continuous variables were presented as mean ± SD while categorical variables were as number (percentage). The independent-samples t-test is used to analyze continuous variables. The χ^2^ test was carried out to evaluate the associations between *KATP* variants and those categorical variables. Binary logistic regression analysis was executed to analyze the associations of *KATP* variants with these types of serum lipid disorder as well as CAS ≥ 50%, Bonferroni correction was carried out to adjust the probability of type I error (false positive). The Cox proportional hazards regression model was carried out to access the crude hazard ratios (HRs) for event free analysis of new-onset IS, adjusted HRs and their 95% confidence intervals (CIs) with corrections for potential covariates. A *P* value < 0.05 is statistically significant. All probabilities are two-tailed.

The differentially expressed (DE) exo-miRs in increased Apo B levels (≥ 80 mg/dL) subjects with different genotypes (GG vs. AA+GA) of rs11046182 were analyzed with edgeR software referring to the criteria of |log2 (Fold Change)| no less than 1 and *P* value less than 0.05. Gene Ontology (GO) category and Kyoto Encyclopedia of Genes and Genomes (KEGG) pathway enrichment were analyzed with the Fisher’s exact test and χ^2^ test, and followed by false discovery rate (FDR) correction. A corrected *P* value < 0.05 was performed to choose significant GO categories and KEGG pathways.

## Supplementary Material

Supplementary Material

Supplementary Figures

Supplementary Tables
