# Supplementary material for: Genetic predisposition and bioinformatics analysis of ATP-sensitive potassium channels polymorphisms with the risks of elevated apolipoprotein B serum levels and its related arteriosclerosis cardiovascular disease
Source: Aging (Albany NY). 2021 Mar 3;13(6):8177–203. doi: 10.18632/aging.202628 (PMC8034914; doi:10.18632/aging.202628)
Supplement: Supplementary Material [file aging-13-202628-s001.pdf]

SUPPLEMENTARY MATERIAL

Supplementary Results

KATP SNPs and genotype frequencies

As shown in Supplementary Table 2, *KATP* SNPs rs1104612 (*P*=0.618) and rs147265929 (*P*=0.470) examined followed to the Hardy-Weinberg equilibrium except rs78148713 and rs145456027 (both *P*<0.001).

Association of *KATP* SNPs with increased TRIG serum levels (≥ 1.7 mmol/L) in study subjects

As shown in Supplementary Table 3, *KATP* SNPs rs11046182 (adjusted OR=0.75, 95%CI: 0.55-1.02, *P*=0.067), rs78148713 (adjusted OR=0.74, 95%CI: 0.33-1.65, *P*=0.463), rs145456027 (adjusted OR=1.26, 95%CI: 0.45-3.55, *P*=0.656) and rs147265929 (adjusted OR=0.59, 95%CI: 0.34-1.02, *P*=0.058) were not associated with increased TRIG serum levels (≥1.7mmol/L).

Association of *KATP* SNPs with increased TC serum levels (≥ 5.2 mmol/L) in study subjects

As shown in Supplementary Table 4, *KATP* SNPs rs11046182 (adjusted OR=0.71, 95%CI: 0.49-1.01, *P*=0.059), rs78148713 (adjusted OR=0.43, 95%CI: 0.17-1.05, *P*=0.064), rs145456027 (adjusted OR=2.00, 95%CI: 0.68-5.89, *P*=0.208) and rs147265929 (adjusted OR=1.25, 95%CI: 0.62-2.48, *P*=0.534) were not associated with increased TC serum levels (≥ 5.2 mmol/L).

Association of *KATP* SNPs with increased LDL-C serum levels (≥ 1.4 mmol/L) in study subjects

As shown in Supplementary Table 5, *KATP* SNPs rs11046182 (adjusted OR=0.92, 95%CI: 0.59-1.44, *P*=0.723), rs78148713 (adjusted OR=0.80, 95%CI: 0.35-1.86, *P*=0.611), rs145456027 (adjusted OR=0.31, 95%CI: 0.06-1.58, *P*=0.160) and rs147265929 (adjusted OR=0.60, 95%CI: 0.23-1.57, *P*=0.295) were not associated with increased LDL-C serum levels (≥ 1.4 mmol/L).

Association of *KATP* SNPs with decreased HDL-C serum levels (< 1.0 mmol/L) in study subjects

As shown in Supplementary Table 6, *KATP* SNPs rs11046182 (adjusted OR=1.10, 95%CI: 0.80-1.52, *P*=0.553), rs78148713 (adjusted OR=1.30, 95%CI: 0.60-2.81, *P*=0.507), rs145456027 (adjusted OR=0.40, 95%CI: 0.15-1.07, *P*=0.067) and rs147265929 (adjusted OR=1.50, 95%CI: 0.83-2.71, *P*=0.181) were not associated with decreased HDL-C serum levels (< 1.0 mmol/L).

Association of *KATP* SNPs with decreased Apo AI serum levels (< 120 mg/dL) in study subjects

As shown in Supplementary Table 7, *KATP* SNPs rs11046182 (adjusted OR=0.87, 95%CI: 0.62-1.24, *P*=0.446), rs78148713 (adjusted OR=0.70, 95%CI: 0.31-1.59, *P*=0.395), rs145456027 (adjusted OR=1.41, 95%CI: 0.45-4.40, *P*=0.551) and rs147265929 (adjusted OR=0.53, 95%CI: 0.26-1.06, *P*=0.073) were not associated with decreased Apo AI serum levels (< 120 mg/dL).

Baseline characteristics of the study subjects with different genotypes of *KATP* rs11046182

As shown in Supplementary Table 8, subjects with GG genotype of *KATP* rs11046182 had higher serum levels of Apo B (*P*<0.001), P2hBS (*P*=0.002) and HsCRP (*P*=0.002) compared to those with AA+GA genotype of the loci.

DE exo-miRs between different genotypes of KATP rs11046182 in subjects with decreased Apo B serum levels (< 80 mg/dL)

Compared to the plasma exo-miRs expression profiling between different genotypes of *KATP* rs11046182 in subjects with increased Apo B serum levels (≥ 80 mg/dL), only 6 of the 41 DE exo-miRs were found to be significantly DE between the two genotypes of rs11046182 in subjects with decreased Apo B serum levels (< 80 mg/dL), as shown in Supplementary Figure 2 and Supplementary Table 9. Among the 6 DE exo-miRs, 2 exo-miRs (miR-31-5p and miR-497-5p) were up-regulated and 4 exo-miRs (miR-320c/d, miR-4429 and miR-134-5p) were down-regulated in subjects carrying GG genotype of rs11046182 compared to those with AA+GA genotype, which exhibited opposite expression patterns in subjects with or without increased Apo B serum levels (≥ 80 mg/dL) under specific genetic background of KATP rs11046182.

Supplementary Method

Carotid and cardiac ultrasonography

Bilateral carotid and cardiac ultrasonic scanning was performed when patients admitted to the study. The near and far walls of bilateral common carotid artery, bifurcations, and 1 cm of the internal and external carotid arteries were scanned for the presence of carotid arteriosclerosis stenosis (CAS) ≥ 50% (recorded as the average of measurements by two independent

experienced physicians according to the measurement of stenosis degree used in the North American Symptomatic Carotid Endarterectomy Trial [1]) with a 3/9 MHz ultra-wideband linear array transducer (iU22, Philips, NL). The left atrial end-diastolic dimension (LAD), left ventricular end-diastolic diameter (LVD), right atrial end-diastolic dimension (RAD), right ventricular end-diastolic diameter (RVD), and left ventricular ejection fraction (LVEF) were measured using M-mode or two-dimensional echocardiography in the parasternal long-axis view at the end-ventricular systole with a 1.7/3.4 MHz linear array transducer (Vivid 7, GE Healthcare, USA) over 4 cardiac cycles according to recommendations for chamber quantification from the American Society of Echocardiography [2].

Sample collection in plasma exosome-derived miRs (exo-miRs) expression profiling analyses

Peripheral venous whole-blood samples were collected into anticoagulation tube with EDTA (3 mg/mL) on enrollment, but after a 12-hours fasting and a light, low-fat meal the night. All tubes were centrifuged within an hour from collection at 3000g (Eppendorf 5810R centrifuge, Germany) for 5 minutes at 4° C to separate plasma and cellular components. Hemolysis was assessed according to the previously reported method, and hemolyzed samples were excluded from the experimental workflow.

Isolation of exosomes from plasma

The upper plasma phase was carefully transferred to a new tube with conical bottom without disturbing the intermediate buffy coat layer and centrifuged for 15 min at 3000 × g for 10 min at 4° C to remove additional cellular fragments and debris. The cleared supernatant was carefully transferred to a new tube without disturbing the pellet, which forms a smear along the bottom of the centrifugation tube. Then, the plasma was passed through a 0.22-μm filter to remove larger extracellular vesicles, aliquoted, and stored at -80° C. Exosomes from prefiltered plasma were isolated with exoEasy Maxi kit (Qiagen, Dusseldorf, Germany; Catalog No. 76064) according to the manufacturer's protocol with modifications described in Stranska et al. [3]. Briefly, 2 ml buffer XBP was added to 2 ml plasma and mixed well by gently inverting the tube five times. Then, the sample/XBP mix was added onto the exoEasy spin column and centrifuged at 500×g for 1 min. The flow-through was discarded and the column was placed back into the same collection tube. After that, 10 ml buffer XWP was added to the column and centrifuged at 3000×g for 5 min to remove any residual buffer from the column. The flow-through along with the collection tube was discarded and the spin column was transferred to a new collection tube. Next, 400 μl buffer XE was added to the membrane and incubated for 1 min, followed by centrifuging at 500×g for 5 min to collect the eluate. Finally, the eluate was re-applied to the exoEasy spin column membrane and incubated for 1 min and then centrifuged at 5000×g for 5 min to collect the eluate.

Extraction RNA from exosomes

Exosomal RNA was extracted by HiPure Liquid miRNA Kit/HiPure Serum/Plasma miRNA Kit (Megan, China) according to the manufacturer's instructions. RNA purity was assessed using NanoDrop-1000 (ThermoFisher, CA, USA). Each RNA sample had an A260:A280 ratio above 1.8 and A260:A230 ratio above 2.0. The quantity and integrity of Exosomal RNA yield was assessed by using the Qubit® 2.0 (Life Technologies, Carlsbad, CA, USA) and Agilent 2200 TapeStation (Agilent Technologies, CA, USA) separately.

Exo-miRs sequencing

Exo-miRs sequencing was performed using Illumina platforms (Illumina, Carlsbad, USA) at Ribobio Co. (Guangzhou, China). Briefly, RNAs (50ng Exosomal RNA of each sample) were ligated with 3'RNA adapter, and followed by 5'adapter ligation. Subsequently, the adapter-ligated RNAs were subjected to RT-PCR and amplified with a low-cycle. Then the PCR products were size selected by PAGE gel according to instructions of NEBNext Multiplex Small RNA Library Prep Set for Illumina (New England Biolabs, MA, USA). The purified Exo-miRs library products were evaluated using the Agilent 2200 TapeStation and diluted to 10 pmol/L for cluster generation in situ on the HiSeq2500 single-end flow cell followed by sequencing (1 × 50 bp) on an Illumina Hiseq 2500 platform. Raw data (raw reads) in fastq format were preprocessed to trim 3' and 5'adapters, and then, the low-quality reads were filtered out to obtain clean reads. By classifying the clean reads, the components and expression information of all types of small RNAs, including miRs, in the sample can be obtained.The miRs expression levels were estimated by the number of reads per million (RPM) using the following formula: RPM=(number of reads mapping to microRNA/number of reads in clean data) $\times 10^6$.

Sequencing data analysis

The raw reads were processed by filtering out containing adapter, poly ' N', low quality, smaller than 17nt reads by FASTQC to get clean reads. Mapping reads were obtained by mapping clean reads to reference genome

of by BWA. The miRDeep2 was used to identify known mature exo-miRs based on miRBase21 (http://www.mirbase.org) and predict novel exo-miRs. Databases of Rfam12.1 (https://rfam.xfam.org) and pirnabank (http://pirnabank.ibab.ac.in) were used to identify rRNA, tRNA, snRNA, snoRNA and piRNA by BLAST. The exo-miRs expression were calculated by RPM (reads per million) values. The expression levels were normalized by RPM, RPM is equal to (number of reads mapping to miRs/number of reads in clean data) $\times 10^6$. Exo-miRs differential expression in subjects with between two genotypes of rs1799858 was calculated by edgeR algorithm according to the criteria of $|\log2 (\text{Fold Change})| \geq 1$ and P value < 0.05.The miRDB, miRTarBase, miRWalk and TargetScan were used to predict targets gene of selected exo-miRs. KOBAS was used to further Gene Ontology (GO) and KEGG (Kyoto Encyclopedia of Genes and Genomes) pathway analysis.

Supplementary References

1. Meschia JF, Klaas JP, Brown RD Jr, Brott TG. Evaluation and Management of Atherosclerotic Carotid Stenosis. Mayo Clin Proc. 2017; 92:1144–57. https://doi.org/10.1016/j.mayocp.2017.02.020 PMID:28688468

2. Lang RM, Badano LP, Mor-Avi V, Afilalo J, Armstrong A, Ernande L, Flachskampf FA, Foster E, Goldstein SA, Kuznetsova T, Lancellotti P, Muraru D, Picard MH, et al. Recommendations for cardiac chamber quantification by echocardiography in adults: an update from the American Society of Echocardiography and the European Association of Cardiovascular Imaging. J Am Soc Echocardiogr. 2015; 28:1–39.e14. https://doi.org/10.1016/j.echo.2014.10.003 PMID:25559473

3. Stranska R, Gysbrechts L, Wouters J, Vermeersch P, Bloch K, Dierickx D, Andrei G, Snoeck R. Comparison of membrane affinity-based method with size-exclusion chromatography for isolation of exosome-like vesicles from human plasma. J Transl Med. 2018; 16:1. https://doi.org/10.1186/s12967-017-1374-6 PMID:29316942
