# Supplementary material for: Genetic predisposition and bioinformatics analysis of ATP-sensitive potassium channels polymorphisms with the risks of elevated apolipoprotein B serum levels and its related arteriosclerosis cardiovascular disease
Source: Aging (Albany NY). 2021 Mar 3;13(6):8177–203. doi: 10.18632/aging.202628 (PMC8034914; doi:10.18632/aging.202628)
Supplement: Supplementary Figures [file aging-13-202628-s002.pdf]

SUPPLEMENTARY FIGURES

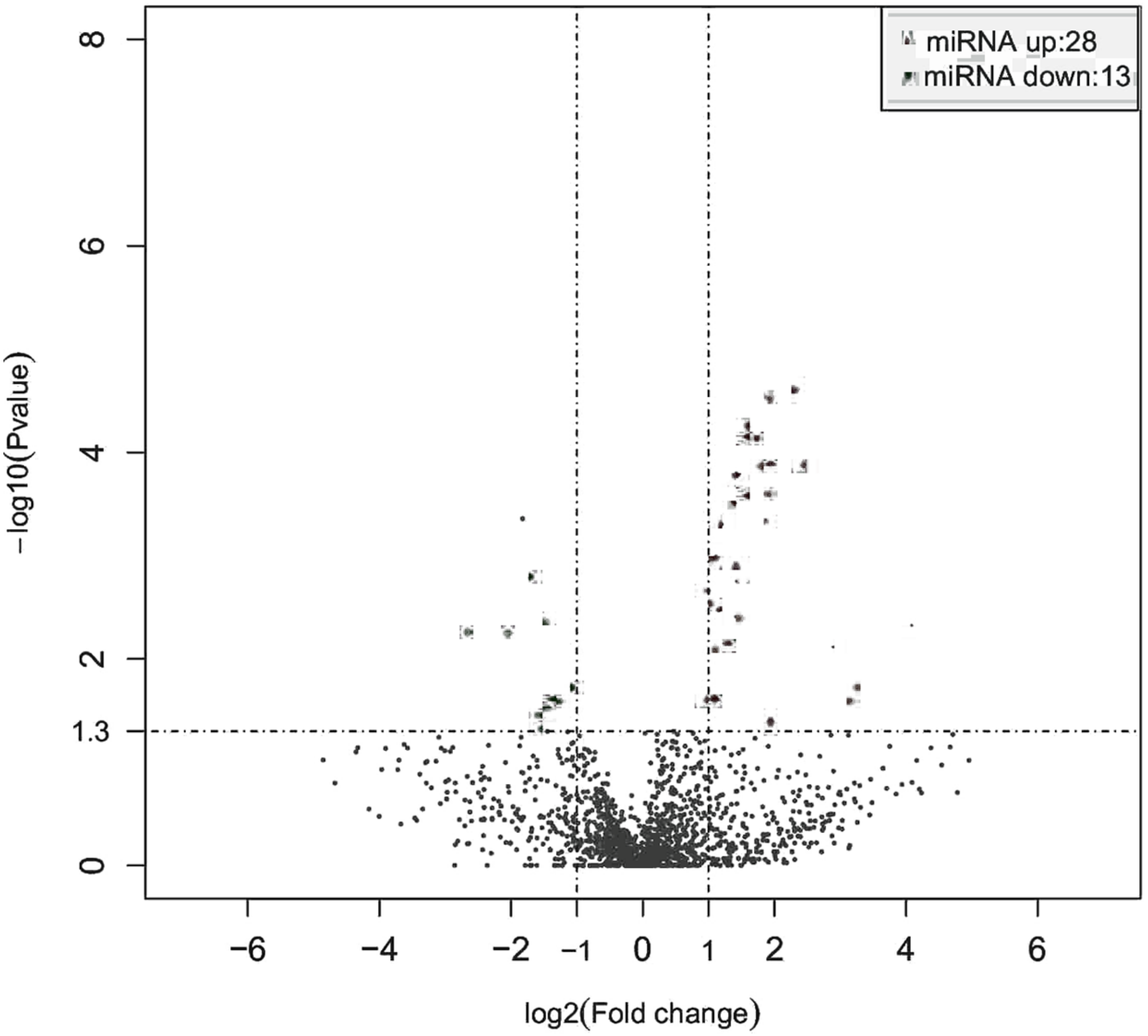

Supplementary Figure 1. Volcano map of DE exo-miRs between different genotypes of *KATP* rs11046182 in subjects with elevated Apo B serum levels (≥ 80 mg/dL).

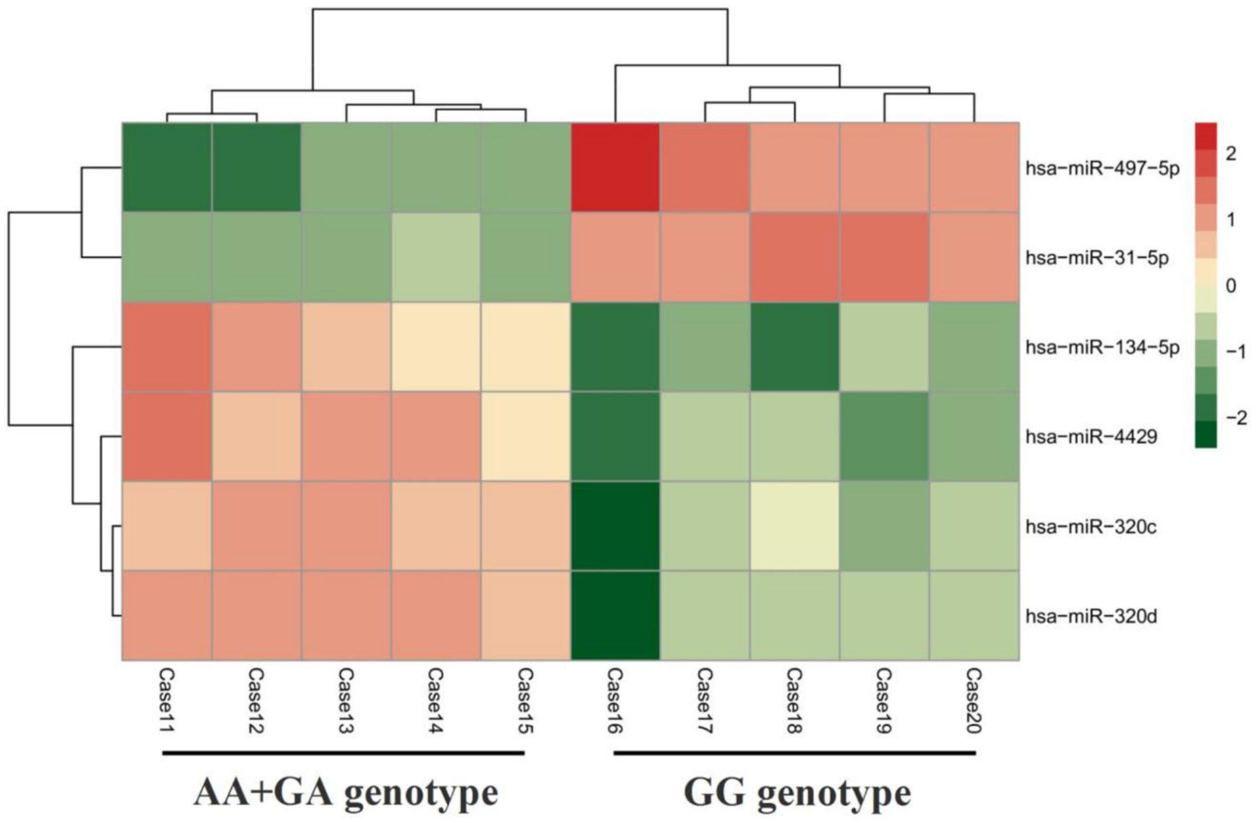

Supplementary Figure 2. Heatmap of DE exo-miRs between different genotypes of *KATP* rs11046182 in subjects with decreased Apo B serum levels (< 80 mg/dL).
