# Supplementary material for: Genetic predisposition and bioinformatics analysis of ATP-sensitive potassium channels polymorphisms with the risks of elevated apolipoprotein B serum levels and its related arteriosclerosis cardiovascular disease
Source: Aging (Albany NY). 2021 Mar 3;13(6):8177–203. doi: 10.18632/aging.202628 (PMC8034914; doi:10.18632/aging.202628)
Supplement: Supplementary Tables [file aging-13-202628-s003.pdf]

SUPPLEMENTARY TABLES

Supplementary Table 1. The primers of KATP SNPs in the sequenom MassARRAY system.

NQ.	SNP_ID	Gene	Protein		Primer
1	*rs11046182*	KCNJ8	Kir 6.1	1st- forward PCR primer (5'-3')	ACGTTGGATGAGATTCTTACAAGGAGCCCG
				2nd- reverse PCR primer (5'-3')	ACGTTGGATGTCTCATAGGAGTGTGAACCC
				extension primer (5'-3')	CCCTACGGTGAACTG
2	*rs78148713*	KCNJ8/ABCC9	Kir 6.1/SUR2	1st- forward PCR primer (5'-3')	ACGTTGGATGAAGTGGAAGCTGCATGAGAG
				2nd- reverse PCR primer (5'-3')	ACGTTGGATGTACTCTTGGGATCTCGGAAC
				extension primer (5'-3')	CCACTCTTGGGATCTCGGAACAATTTG
3	*rs145456027*	KCNJ8/ABCC9	Kir 6.1/SUR2	1st- forward PCR primer (5'-3')	ACGTTGGATGCAAAGCTGTAGGCATCACAC
				2nd- reverse PCR primer (5'-3')	ACGTTGGATGGTACCAGTACCTTGCTGTTC
				extension primer (5'-3')	GGCTTTTCTGGTTACTGTAGCCTTGTAG
4	*rs147265929*	KCNJ11/ABCC8	Kir 6.2/SUR1	1st- forward PCR primer (5'-3')	ACGTTGGATGTTCCTTTCCGAGCTTCTCTG
				2nd- reverse PCR primer (5'-3')	ACGTTGGATGAGAAAAGCCCACCAGTTATC
				extension primer (5'-3')	GAGCGGCCCACCAGTTATCGGAGGC

Supplementary Table 2. Descriptive information on *KATP* SNPs in study subjects.

NQ	KATP SNPs	MAF in CHB	Major/minor allele	MAF*		P_{HWE}-value#	Power
				Apo B < 80 mg/dL	Apo B ≥ 80 mg/dL		
1	*rs11046182*	0.180	*G/A*	0.247	0.196	0.618	0.999
2	*rs78148713*	0.053	*T/C*	0.038	0.017	<0.001	0.124
3	*rs145456027*	0.063	*T/C*	0.025	0.011	<0.001	0.388
4	*rs147265929*	0.053	*T/G*	0.012	0.048	0.470	0.698

*MAF: minor allele frequency; CHB: Han Chinese in Beijing, China; #P_{HWE} value for subjects with Apo B<80mg/dL(control).

Supplementary Table 3. Association of *KATP* SNPs with increased TRIG serum levels (≥ 1.7 mmol/L) in study subjects.

KATP SNPs		TRIG ≥ 1.7 mmol/L (N/%)		$\chi 2$	*P* value	Crude OR (95% CI)	Crude *P* value	Adjusted OR (95% CI)*	Adjusted *P* value*	Adjusted OR (95% CI)#	Adjusted *P* value#
		NO	YES								
rs11046182	*GG*	445(62.8)	198(59.1)	1.288	0.256	0.86(0.66-1.12)	0.257	0.77(0.58-1.03)	0.078	0.75(0.55-1.02)	0.067
	AA+GA	264(37.2)	137(40.9)			1.00		1.00		1.00	
rs78148713	*CC+CT*	34(4.8)	10(3.0)	1.847	0.174	0.61(0.30-1.25)	0.178	0.60(0.29-1.27)	0.184	0.74(0.33-1.65)	0.463
	TT	675(95.2)	325(97.0)			1.00		1.00		1.00	
rs145456027	*CC+CT*	16(2.3)	8(2.4)	0.017	0.895	1.00		1.00		1.00	
	TT	508(97.7)	335(97.6)			0.94(0.40-2.23)	0.895	1.00(0.41-2.45)	0.996	1.26(0.45-3.55)	0.656
rs147265929	*GG+GT*	46(6.5)	32 (9.6)	3.090	0.079	1.00		1.00		1.00	
	TT	663(93.5)	303(90.4)			0.66(0.41-1.05)	0.081	0.62(0.37-1.04)	0.069	0.59(0.34-1.02)	0.058

*Model 1: After adjustment for gender, age, smoking, drinking, WBC, BMI, EH, T2D, liver function (ALT, AST and Alb), renal function (Scr, BUN and UA), HsCRP, HbA1C, HCY, and RAAS activity (ACE, renin, Ang I, Ang II and ALD).
#Model 2b: It is the same as Model 1, and also including dyslipidemia (TC, LDL-C, Apo B, HDL-C and Apo AI).

Supplementary Table 4. Association of *KATP* SNPs with increased TC serum levels (≥ 5.2 mmol/L) in study subjects.

KATP SNPs		TC ≥ 5.2 mmol/L (N/%)		χ2	*P* value	Crude OR (95% CI)	Crude *P* value	Adjusted OR (95% CI)*	Adjusted *P* value*	Adjusted OR (95% CI)#	Adjusted *P* value#
		NO	YES								
rs11046182	*GG*	296(62.7)	347(60.7)	0.458	0.498	0.92(0.71-1.18)	0.498	0.95(0.73-1.24)	0.729	0.71(0.49-1.01)	0.059
	AA+GA	176(37.3)	225(39.3)			1.00				1.00	
rs78148713	*CC+CT*	30(6.4)	14(2.4)	9.785	0.002	0.37(0.19-0.71)	0.003	0.32(0.17-0.63)	0.001	0.43(0.17-1.05)	0.064
	TT	442(93.6)	558(97.6)			1.00		1.00		1.00	
rs145456027	*CC+CT*	14(3.0)	10(1.7)	1.708	0.191	1.00		1.00		1.00	
	TT	458(97.0)	562(98.3)			1.72(0.76-3.90)	0.196	2.22(0.95-5.18)	0.065	2.00(0.68-5.89)	0.208
rs147265929	*GG+GT*	34(7.2)	44(7.7)	0.089	0.765	1.00		1.00		1.00	
	TT	438(92.8)	528(92.3)			0.93(0.59-1.48)	0.765	0.91(0.56-1.49)	0.707	1.25(0.62-2.48)	0.534

*Model 1: After adjustment for gender, age, smoking, drinking, WBC, BMI, EH, T2D, liver function (ALT, AST and Alb), renal function (Scr, BUN and UA), HsCRP, HbA1C, HCY, and RAAS activity (ACE, renin, Ang I, Ang II and ALD).
#Model 2d: It is the same as Model 1, and also including dyslipidemia (TRIG, LDL-C, Apo B, HDL-C and Apo AI).

Supplementary Table 5. Association of *KATP* SNPs with increased LDL-C serum levels (≥ 1.4 mmol/L) in study subjects.

KATP SNPs		LDL-C ≥ 1.4 mmol/L (N/%)		χ2	*P* value	Crude OR (95% CI)	Crude *P* value	Adjusted OR (95% CI)*	Adjusted *P* value*	Adjusted OR (95% CI)#	Adjusted *P* value#
		NO	YES								
rs11046182	*GG*	84(60.9)	559(61.7)	0.035	0.852	1.04(0.72-1.50)	0.852	1.00(0.68-1.47)	0.978	0.92(0.59-1.44)	0.723
	AA+GA	54(37.2)	347(38.3)			1.00		1.00		1.00	
rs78148713	*CC+CT*	10(7.2)	34(3.8)	3.621	0.057	0.50(0.24-1.04)	0.062	0.48(0.22-1.02)	0.057	0.80(0.35-1.86)	0.611
	TT	128(92.8)	872(96.2)			1.00		1.00		1.00	
rs145456027	*CC+CT*	2(1.4)	22(2.4)	0.511	0.475	1.00		1.00		1.00	
	TT	136(98.6)	884(97.6)			0.59(0.14-2.54)	0.480	0.58(0.13-2.65)	0.486	0.31(0.06-1.58)	0.160
rs147265929	*GG+GT*	8(5.8)	70(7.7)	0.645	0.422	1.00		1.00		1.00	
	TT	130(94.2)	836(92.3)			0.74(0.35-1.56)	0.424	0.63(0.27-1.46)	0.276	0.60(0.23-1.57)	0.295

*Model 1: After adjustment for gender, age, smoking, drinking, WBC, BMI, EH, T2D, liver function (ALT, AST and Alb), renal function (Scr, BUN and UA), HsCRP, HbA1C, HCY, and RAAS activity (ACE, renin, Ang I, Ang II and ALD).
#Model 2c: It is the same as Model 1, and also including dyslipidemia (TRIG, TC, Apo B, HDL-C and Apo AI).

Supplementary Table 6. Association of *KATP* SNPs with decreased HDL-C serum levels (< 1.0 mmol/L) in study subjects.

KATP SNPs		HDL-C <1.0mmol/L (N/%)		χ2	*P* value	Crude OR (95% CI)	Crude *P* value	Adjusted OR (95% CI)*	Adjusted *P* value*	Adjusted OR (95% CI)#	Adjusted *P* value#
		NO	YES								
rs11046182	*GG*	391(60.9)	252(62.7)	0.332	0.564	1.08(0.83-1.39)	0.564	1.02(0.77-1.35)	0.892	1.10(0.80-1.52)	0.553
	AA+GA	251(39.1)	150(37.3)			1.00		1.00		1.00	
rs78148713	*CC+CT*	24(3.7)	20(5.0)	0.937	0.333	1.35(0.74-2.47)	0.335	1.54(0.81-2.92)	0.187	1.30(0.60-2.81)	0.507
	TT	618(96.3)	382(95.0)			1.00		1.00		1.00	
rs145456027	*CC+CT*	12(3.0)	12(3.0)	1.371	0.242	1.00		1.00		1.00	
	TT	630(98.1)	390(97.0)			0.62(0.28-1.39)	0.246	0.34(0.14-0.81)	0.015	0.40(0.15-1.07)	0.067
rs147265929	*GG+GT*	52(8.1)	26(6.5)	0.952	0.329	1.00		1.00		1.00	
	TT	590(91.9)	376(93.5)			1.28(0.78-2.08)	0.330	1.17(0.69-1.98)	0.555	1.50(0.83-2.71)	0.181

*Model 1: After adjustment for gender, age, smoking, drinking, WBC, BMI, EH, T2D, liver function (ALT, AST and Alb), renal function (Scr, BUN and UA), HsCRP, HbA1C, HCY, and RAAS activity (ACE, renin, Ang I, Ang II and ALD).
#Model 2e: It is the same as Model 1, and also including dyslipidemia (TRIG, TC, LDL-C, Apo B and Apo AI).

Supplementary Table 7. Association of *KATP* SNPs with decreased Apo AI serum levels (< 120 mg/dL) in study subjects.

KATP SNPs		Apo AI <120 mg/dL (N/%)		χ2	*P* value	Crude OR (95% CI)	Crude *P* value	Adjusted OR (95% CI)*	Adjusted *P* value*	Adjusted OR (95% CI)#	Adjusted *P* value#
		NO	YES								
rs11046182	*GG*	172(61.9)	471(61.5)	0.013	0.911	0.98(0.74-1.31)	0.911	0.94(0.69-1.27)	0.670	0.87(0.62-1.24)	0.446
	AA+GA	106(38.1)	295(38.5)			1.00				1.00	
rs78148713	*CC+CT*	12(4.3)	32(4.2)	0.010	0.921	0.97(0.49-1.90)	0.921	1.11(0.54-2.28)	0.779	0.70(0.31-1.59)	0.395
	TT	266(95.7)	734(95.8)			1.00		1.00		1.00	
rs145456027	*CC+CT*	6(2.2)	18(2.3)	0.033	0.855	1.00		1.00		1.00	
	TT	272(97.8)	748(97.7)			0.92(0.36-2.33)	0.855	0.74(0.27-2.03)	0.559	1.41(0.45-4.40)	0.551
rs147265929	*GG+GT*	14(5.0)	64(8.4)	3.250	0.071	1.00		1.00		1.00	
	TT	264(95.0)	702(91.6)			0.58(0.32-1.06)	0.074	0.63(0.34-1.19)	0.157	0.53(0.26-1.06)	0.073

*Model 1: After adjustment for gender, age, smoking, drinking, WBC, BMI, EH, T2D, liver function (ALT, AST and Alb), renal function (Scr, BUN and UA), HsCRP, HbA1C, HCY, and RAAS activity (ACE, renin, Ang I, Ang II and ALD).
#Model 2f: It is the same as Model 1, and also including dyslipidemia (TRIG, TC, LDL-C, Apo B and HDL-C).

Supplementary Table 8. Baseline characteristics of the study subjects with different genotypes of *KATP* rs11046182.

	Genotypes of KATP rs11046182 (N/%)		*P* value
	GG	AA+GA	
N	643	401	-
Male: Female	506:137	295:106	0.057
Age (Y)	64.2±10.8	64.6±11.3	0.551
Smoking (%)	349(54.3)	197(49.1)	0.105
Drinking (%)	99(15.4)	51(12.7)	0.230
SBP (mmHg)	138.5±22.7	138.2±23.2	0.824
DBP (mmHg)	78.4±13.2	77.9±11.1	0.536
BMI (kg/m^2)	24.5±3.9	24.9±4.5	0.164
**Medical condition**			
EH (%)	411(63.9)	245(61.1)	0.359
CHD (%)	509(79.2)	323(80.5)	0.588
T2D (%)	330(52.9)	195(46.1)	0.397
AF (%)	28(4.4)	10(2.5)	0.118
**Blood biochemical index**			
TRIG (mmol/L)	1.51±0.87	1.59±0.94	0.133
TC (mmol/L)	4.25±1.26	4.32±1.17	0.386
HDL-C (mmol/L)	1.09±0.29	1.10±0.25	0.708
LDL-C (mmol/L)	2.33±0.85	2.34±0.85	0.738
Apo A1 (mg/dL)	104.8±24.1	105.7±24.8	0.583
**Apo B (mg/dL)**	**93.3±27.4**	**81.2±33.8**	**<0.001**
WBC (×10^9/L)	8.52±3.00	8.35±2.70	0.355
HGB (g/L)	131.5±17.9	132.8±18.2	0.288
PLT (×10^9/L)	232.4±62.2	235.7±67.7	0.428
FBG (mmol/L)	5.66±1.39	5.50±1.32	0.061
**P2hBS (mmol/L)**	**9.12±2.97**	**8.56±2.62**	**0.002**
HbA1C (%)	6.0±1.3	5.9±1.2	0.091
Cr (μmol/L)	90.7±42.1	91.8±32.9	0.632
BUN (mmol/L)	5.76±1.81	5.75±1.74	0.980
UA (μmol/L)	406.8±111.1	417.6±111.2	0.126

ALT (U/L)	28.4±28.6	29.9±18.8	0.335
AST (U/L)	48.0±59.1	49.6±59.4	0.684
Alb (g/L)	37.2±4.3	37.4±3.4	0.438
Na^+ (mmol/L)	140.3±3.1	140.6±3.3	0.172
K^+ (mmol/L)	3.75±0.40	3.71±0.40	0.163
**HsCRP (mg/L)**	**14.3±24.3**	**10.2±13.0**	**0.002**
ACE (U/L)	34.9±20.4	32.5±22.5	0.082
Renin (pg/mL)	25.2±28.2	25.9±29.6	0.709
Ang I (ng/L)	2.17±1.68	2.14±1.53	0.791
Ang II (ng/L)	66.8±97.2	69.2±93.4	0.692
ALD (ng/L)	180.6±103.4	180.8±107.1	0.974
**Echocardiography**			
**RVD (cm)**	1.74±0.19	1.75±0.17	0.590
**RAD (cm)**	3.36±0.36	3.34±0.23	0.313
**LVD (cm)**	4.82±0.56	4.80±0.56	0.495
**LAD (cm)**	3.13±0.55	3.07±0.58	0.101
**LVEF (%)**	56.7±9.8	56.6±8.8	0.787

Supplementary Table 9. DE exo-miRs between different genotypes of *KATP* rs11046182 in subjects with decreased Apo B serum levels (< 80 mg/dL).*

	miR ID	Genotypes		Fold	*P* value	Up/down
		AA+GA	GG			
1	**hsa-miR-31-5p**	**1.01**	**8.60**	**3.03**	**0.002392**	**Up**
2	hsa-miR-451b	6.56	3.47	-0.86	0.366050	NS
3	hsa-miR-499a-5p	58.36	96.44	0.73	0.352732	NS
4	hsa-miR-671-3p	374.86	183.18	-1.04	0.177117	NS
5	hsa-miR-208b-3p	2.08	4.33	1.06	0.399135	NS
6	hsa-miR-937-3p	5.33	10.94	1.04	0.138973	NS
7	hsa-miR-493-5p	82.20	206.72	1.33	0.084805	NS
8	hsa-miR-208a-3p	3.04	1.15	-1.24	0.296911	NS
9	hsa-miR-218-5p	24.25	70.77	1.55	0.071521	NS
10	hsa-miR-1298-5p	11.05	2.81	-1.95	0.061239	NS
11	**hsa-miR-497-5p**	**1.16**	**52.90**	**5.42**	**0.000834**	**Up**
12	hsa-miR-4661-5p	29.75	23.15	-0.38	0.687832	NS
13	hsa-miR-943	3.33	3.18	-0.02	1.000000	NS
14	hsa-miR-490-3p	4.63	2.29	-1.02	0.064719	NS
15	hsa-miR-378c	168.86	57.06	-1.57	0.096213	NS
16	hsa-miR-378g	39.08	61.38	0.65	0.301826	NS
17	hsa-miR-378f	47.41	71.04	0.59	0.330965	NS
18	hsa-miR-1291	14.57	20.68	0.49	0.511690	NS
19	hsa-miR-378e	4.67	13.86	1.55	0.094618	NS
20	hsa-miR-378h	1.73	3.41	0.92	0.262292	NS
21	hsa-miR-378i	943.52	999.31	0.08	0.869687	NS
22	hsa-miR-378a-3p	14746.92	13642.93	-0.11	0.805230	NS
23	**hsa-miR-320d**	**2968.60**	**1220.93**	**-1.28**	**0.025454**	**Down**
24	hsa-miR-422a	28.56	28.71	0.01	1.000000	NS
25	hsa-miR-378d	201.36	431.17	1.10	0.061050	NS
26	hsa-miR-378b	3.30	4.35	0.52	0.644108	NS
27	hsa-miR-22-3p	4874.83	2056.63	-1.25	0.051735	NS
28	**hsa-miR-4429**	**125.80**	**39.32**	**-1.69**	**0.009841**	**Down**
29	hsa-miR-320e	518.56	242.54	-1.10	0.107776	NS
30	hsa-miR-4726-5p	2.95	4.53	0.56	0.587542	NS
31	hsa-miR-7704	2.72	2.65	-0.08	1.000000	NS
32	hsa-miR-210-3p	41.63	15.68	-1.41	0.050443	NS
33	**hsa-miR-320c**	**9222.73**	**3050.18**	**-1.60**	**0.002334**	**Down**
34	**hsa-miR-134-5p**	**5900.22**	**1763.79**	**-1.74**	**0.005120**	**Down**
35	hsa-miR-4488	3.78	5.55	0.50	0.606224	NS
36	hsa-miR-3960	5.71	3.10	-1.00	0.309417	NS
37	hsa-miR-193a-5p	3454.30	1373.34	-1.33	0.127135	NS
38	hsa-miR-551b-5p	4.84	2.61	-1.03	0.431600	NS
39	hsa-miR-193b-5p	7.97	3.88	-1.04	0.095301	NS
40	hsa-miR-17-3p	1.82	0.77	-1.24	0.075457	NS
41	hsa-miR-4497	2.39	1.12	-1.11	0.369703	NS

*NS: no significant difference.
